# A Hidden Complication of Poorly Managed Diabetic Control: A Case of Diabetic Myonecrosis

**DOI:** 10.7759/cureus.43210

**Published:** 2023-08-09

**Authors:** Tejaswini Takkellapati, Vamsi Krishnamaneni, Maneeth Mylavarapu, Rohil Krishna Bobba, Rathagna Kundakarla

**Affiliations:** 1 Internal Medicine, Mamata Medical College, Khammam, IND; 2 Internal Medicine, Katuri Medical College, Guntur, IND; 3 Public Health, Adelphi University, Garden City, USA; 4 Internal Medicine, Dr. D. Y. Patil Vidyapeeth, Pune, IND

**Keywords:** primary muscle disease, muscle infarction, glycemic control, diabetes mellitus, diabetic myonecrosis

## Abstract

Diabetic myonecrosis is an infrequently encountered complication of poorly managed type 2 diabetes. Despite its relative rarity, early detection and appropriate management can yield favorable outcomes. This case report details the presentation, diagnosis, and management of a 53-year-old male patient with a history of type 2 diabetes who presented with acute-onset pain, swelling of the muscles, and weakness. Following a battery of laboratory investigations, radiological imaging, and a muscle biopsy, the patient was diagnosed with diabetic myonecrosis. The patient was treated conservatively with analgesics, physiotherapy, and optimization of glycemic control, significantly improving muscle strength and function. This case highlights the necessity of considering diabetic myonecrosis as a potential differential diagnosis in diabetic patients who present with sudden muscle weakness and discomfort.

## Introduction

Type 2 diabetes, a metabolic disease with a global prevalence of 8.7%, presents a significant health burden worldwide [1]. The disease's prevalence in India notably increased from 7.1% in 2009 to 8.9% in 2019 [2]. This form of diabetes is associated with numerous microvascular and macrovascular complications, including diabetic myonecrosis. Also known as diabetic muscle infarction, this unusual microvascular complication of poorly controlled type 2 diabetes was first identified by Angervall et al. in 1965 as tumoriform focal muscle infarction [3]. Despite its uncommonness, diabetic myonecrosis generally responds well to conservative treatment and proper glycemic control. In this case report, we discuss a 53-year-old male patient with a history of type 2 diabetes who presented with acute pain, muscle swelling, and weakness, ultimately diagnosed with diabetic myonecrosis.

## Case presentation

A 53-year-old male from Pimpri, Pune, reported bilateral upper and lower limb weakness that had progressively worsened over eight days. This sudden-onset weakness was most pronounced in the proximal muscles, hindering the patient's ability to stand from a squatting position, climb stairs, and perform simple tasks such as combing his hair. Over time, distal muscle weakness also developed, leading to difficulties in bed mobility. Additionally, the patient experienced bilateral pain and swelling in his hands and legs over the same period. Despite a 10-year history of diabetes, he had stopped taking his prescribed oral hypoglycemic medications for the past 20 days.

On examination, his blood pressure was 110/80 mmHg, with a pulse rate of 84 beats per minute. We observed significant pitting edema in the affected areas, accompanied by induration and tenderness. All muscle groups displayed tenderness, and both passive and active joint movements were limited due to pain. Reflexes were diminished, with biceps and triceps reflexes on both sides graded as 1+, bilateral knee reflex graded as 1+, and absent ankle reflexes with bilateral plantar flexors.

Upon admission, the patient's blood glucose level was significantly elevated at 530 mg/dL, with a large presence of ketones in the urine. His total leukocyte count was 7600, and he had elevated C-reactive protein levels (88 mg/dL). His erythrocyte sedimentation rate was slightly elevated at 22 mm/h, creatine phosphokinase levels were significantly increased at 13,122 U/L, and lactate dehydrogenase levels were 867 U/L. His autoimmune screening showed negative results. His glycated hemoglobin level was 9.6%, indicating poor glycemic control. Urinalysis revealed the presence of glucose, proteinuria (+1), and acetones, but renal function tests and serum electrolytes were within reference limits. Ultrasonography of the abdomen and pelvis showed no nephropathic changes, and a fundus examination revealed no retinopathic changes.

**Figure 1:**
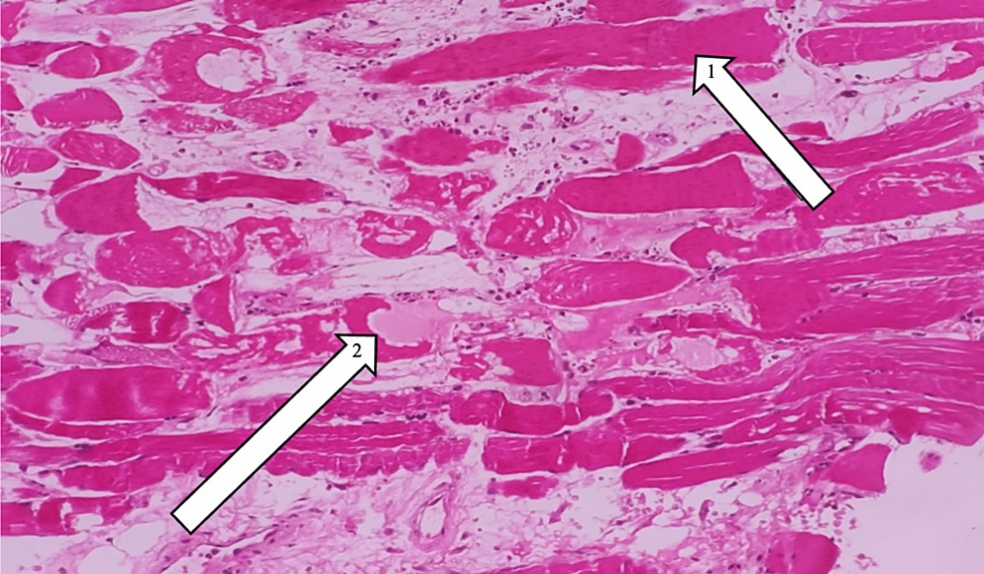
Histopathology image of thigh muscle biopsy. (1) Necrotic muscle fibers (swollen and eosinophilic and lack striations and nuclei). (2) Infarcted patch of myocytes.

We performed electromyography with a nerve conduction velocity study to evaluate for neuropathy and myopathic changes. The results indicated primary muscle disease without any axonal or degenerative changes. A subsequent muscle biopsy revealed individual muscle fibers' necrosis surrounded by macrophages (Figure 1). These fibers exhibited loss of striations, cytoplasmic eosinophilia, and karyorrhexis, with evidence of muscle fiber regeneration characterized by basophilic cytoplasm and prominent nucleoli. These findings suggested prominent myonecrosis with foci of regeneration.

We diagnosed the patient with diabetic myonecrosis based on his history, clinical features, laboratory results, and biopsy findings. Initial treatment included antibiotics for possible infective myositis and a trial of steroids for potential primary muscle disease. We managed his condition conservatively with analgesics and physiotherapy, optimizing his glycemic control with human insulin and glargine. This approach led to a significant improvement in muscle strength and function.

## Discussion

Diabetic myonecrosis is a rare complication of uncontrolled and long-standing type 2 diabetes. The precise pathophysiology of diabetic myonecrosis is not entirely understood, but it is believed to involve vasculopathy changes due to long-standing poorly controlled diabetes, vasculitis changes, hypercoagulability, and ischemia-reperfusion injury. The process is thought to start with endothelial damage, leading to tissue ischemia, which triggers an inflammatory cascade resulting in ischemic necrosis. The subsequent reperfusion of necrotic tissues generates reactive oxygen species and inflammatory mediators like tumor necrosis and platelet-activating factors, contributing to vasculopathy changes [4]. Changes in the coagulation-fibrinolysis system may also promote hypercoagulability and vascular endothelial damage [5].

Typically, diabetes myonecrosis presents as sudden-onset pain in the lower limbs, commonly affecting the thigh and calf muscles [6]. Although most cases are unilateral, bilateral presentations, like the one observed in our patient, can occasionally occur. A similar case was reported by Cumberledge et al., where the patient presented with left lower limb pain and swelling, with no history of fever and chills, but also had complicating factors of peripheral neuropathy, retinopathy, nephropathy, and gastroparesis. The diagnosis of diabetic myonecrosis was made with the help of MRI, and the patient was managed with analgesics, rest, and strict glycemic control [7]. Nephropathy (71% of cases), neuropathy (55% of cases), and retinopathy (57% of cases) are often concurrent with diabetes myonecrosis [8].

In the patient reported by Cumberledge et al., the diagnosis of diabetic myonecrosis was facilitated by the patient's existing complications. However, our case was not as straightforward, as the presentation was atypical, leading us to consider a biopsy without a prior MRI. Our case emphasizes the importance of considering diabetic myonecrosis as a possible complication in diabetic patients, even in the absence of typical organ involvement, such as nephropathy, neuropathy, or retinopathy, even in poorly controlled diabetes.

A high index of suspicion is necessary for clinicians to diagnose this condition when a patient presents with non-traumatic muscular pain coupled with clinical examination findings, laboratory results, and imaging data. While the short-term prognosis is good, the long-term outlook remains poor due to a high recurrence rate. Strict glycemic control is vital to prevent recurrences.

## Conclusions

We described the symptoms, diagnosis, and treatment of a 53-year-old male patient with a history of type 2 diabetes who came forward with sudden-onset pain, muscle swelling, and weakness. After comprehensive laboratory tests, radiological imaging, and a muscle biopsy, the patient was diagnosed with diabetic myonecrosis. This case underscores the importance of comprehensive diabetic care, encompassing adherence to treatment plans and regular check-ups to achieve optimal glucose control. It also highlights the need to consider diabetic myonecrosis as a possible differential diagnosis in diabetic patients presenting with sudden muscle weakness and discomfort.

## References

[1] (2023). Diabetes statistics. https://www.niddk.nih.gov/health-information/health-statistics/diabetes-statistics.

[2] Pradeepa R, Mohan V (2021). Epidemiology of type 2 diabetes in India. Indian J Ophthalmol.

[3] Angervall L, Stener B (1965). Tumoriform focal muscular degeneration in two diabetic patients. Diabetologia.

[4] Rocca PV, Alloway JA, Nashel DJ (1993). Diabetic muscular infarction. Semin Arthritis Rheum.

[5] Carden DL, Granger DN (2000). Pathophysiology of ischaemia-reperfusion injury. J Pathol.

[6] Choudhury BK, Saikia UK, Sarma D, Saikia M, Choudhury SD, Bhuyan D (2011). Diabetic myonecrosis: an underreported complication of diabetes mellitus. Indian J Endocrinol Metab.

[7] Cumberledge J, Kumar B, Rudy D (2016). Risking life and limb: a case of spontaneous diabetic muscle infarction (diabetic myonecrosis). J Gen Intern Med.

[8] Trujillo-Santos AJ (2003). Diabetic muscle infarction: an underdiagnosed complication of long-standing diabetes. Diabetes Care.

